# Effect of phytosterols and inulin-enriched soymilk on LDL-cholesterol in Thai subjects: a double-blinded randomized controlled trial

**DOI:** 10.1186/s12944-015-0149-4

**Published:** 2015-11-09

**Authors:** Noppadol Kietsiriroje, Jirateep Kwankaew, Sunita Kitpakornsanti, Rattana Leelawattana

**Affiliations:** Division of Endocrinology and Metabolism, Department of Internal Medicine, Faculty of Medicine, Prince of Songkla University, 15 Kanjanavanish Rd, Hat Yai, Songkhla 90110 Thailand; Internal Medicine Clinic, Samitivej Srinakarin Hospital, Bangkok, 10250 Thailand; Division of Internal Medicine, Trang Hospital, Trang, 92000 Thailand

**Keywords:** Phytosterols, Inulin, LDL-c, Lipid profile

## Abstract

**Background:**

Hypercholesterolemia, particularly high LDL-c and non-HDL-c levels, is a traditional risk for cardiovascular disease. Ingestion of diets containing phytosterols and inulin can reduce plasma LDL-c and triglyceride levels, respectively. Phytosterols and inulin-enriched soymilk may be an alternative for a supplemental diet to improve both LDL-c and non-HDL-c to reduce the risk of cardiovascular disease.

**Methods:**

Two hundred and forty subjects who were 18 years old or older and had a baseline LDL-c of 130 mg/dl or higher were enrolled into the double-blinded randomized controlled trial study. Subjects were randomly assigned into the study group that received 2 g/day of phytosterols and 10 g/day of inulin-enriched soymilk or into the control group that received standard soymilk. The lipid profile was measured every 2 weeks for 8 weeks. Primary outcomes were 1) to determine the LDL-c reduction after consumption of phytosterols and inulin-enriched soymilk for 8 weeks and 2) to compare the difference of the LDL-c levels between the study and control groups. The secondary outcomes were to compare the difference of TC, TG and HDL-c between the study and control groups.

**Results:**

At the end of the study, the median LDL-c levels decreased significantly from 165 (132, 254) mg/dl to 150 (105, 263) mg/dl in the study group (*p* < 0.001) and from 165 (130, 243) mg/dl to 159 (89, 277) mg/dl in the control group (*p* = 0.014). The LDL-c reduction was significantly better in the study group (−10.03 %, (−37.07, 36.00) vs −1.31 % (−53.40, 89.73), *p* < 0.001). TC also reduced significantly by 6.60 % in the study group while it reduced only by 1.76 % in the control group (*p* < 0.001). There were no statistical differences in TG and HDL-c levels between both study groups. The adverse events in the study group and the control groups were not different (RR 1.33 [0.871-2.030, 95 % CI]).

**Conclusion:**

Daily consumption of soymilk containing 2 g of phytosterols and 10 g of inulin reduced TC and LDL-c better than standard soymilk. It had no effect on TG and HDL-c levels compared to standard soymilk. Both soymilk products were comparably safe.

**Trial registration:**

Thai Clinical Trial Registry: TCTR20150417001 date: April 17, 2015

**Electronic supplementary material:**

The online version of this article (doi:10.1186/s12944-015-0149-4) contains supplementary material, which is available to authorized users.

## Background

**Fig. 1:**
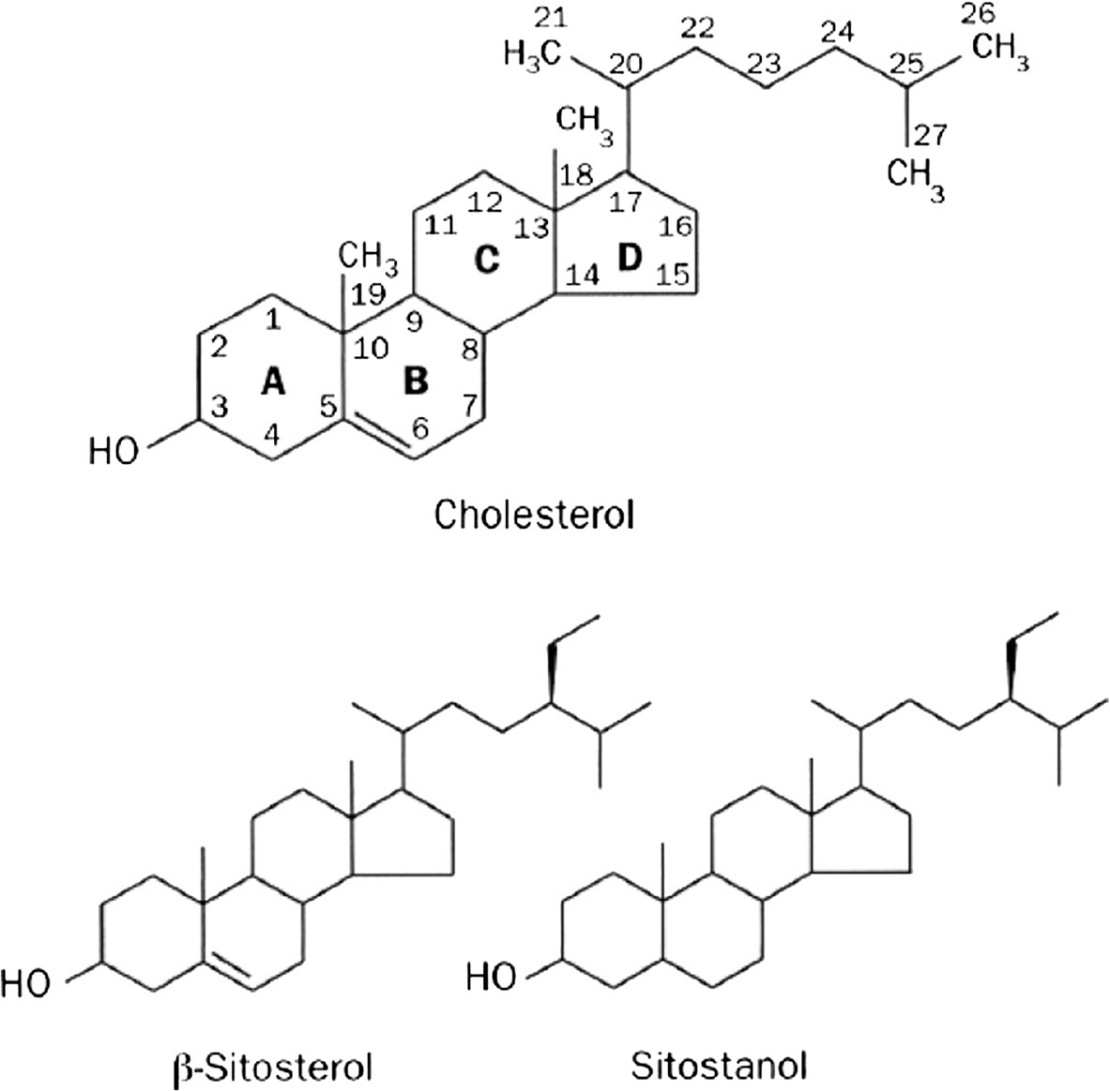
Structure of phytosterols (sitosterol and sitostanol)

Hypercholesterolemia, particularly low-density lipoprotein cholesterol (LDL-c), is an important risk factor for cardiovascular disease. In addition to lipid-lowering agents, recent guidelines have suggested to intake a diet which includes 2 g/day of phytosterols including plant sterol and plant stanol to decrease LDL-c [1–4]. Phytosterols are sterols from plants which are unable to be synthesized by the human body, thus it is necessary to add them (e.g., nuts, legumes and seeds) to the diet [5]. The structure of phytosterols is similar to cholesterol [6] (Fig. 1) and they compete with the cholesterol to incorporate into mixed micelles formation inside the intestinal lumen. Finally micelles containing phytosterols are absorbed into the enterocytes through the Niemann-Pick C1-like 1 (NPC1L1) dependent pathway but phytosterols will efflux back into the intestinal lumen through heterodimeric sterol transporter ABCG5/ABCG8 [ATP-binding cassette (ABC) transporters G5 and G8] [7]. With this process, the ingestion of 2–2.5 g/day of phytosterols in the diet can reduce plasma LDL-c levels by 8-15 % in patients with or without a concomitant statin [8, 9]. From a meta-analysis, the LDL-c reduction effect depended on the amount of phytosterols ingested and the effect was sustained after the intake of 2 g or more per day of phytosterols [10]. Phytosterols have a neutral effect on the triglyceride (TG) or high-density lipoprotein cholesterol (HDL-c) levels in plasma [11]. There is a lack of data on phytosterols content in the Thai diet which should be lower than the western diet that usually contains 150–450 mg of phytosterols per day; however, the total amount of phytosterols in the western diet is inadequate according to recent guideline recommendations. Phytosterols-enriched soymilk is an alternative method to increase the total phytosterols intake to meet the adequate amount recommended in the guidelines.

A relationship between the TG level and the incidence of cardiovascular disease (CVD) is still controversial but there was evidence of stronger links between hypertriglyceridemia and CVD risk in people with lower levels of HDL-c and LDL-c [12, 13] and with type 2 diabetes mellitus (T2DM) [14]. The National Lipid Association (NLA) Expert Panel recommendations also indicated that the normal level of serum TG was less than 150 mg/dl [15].

Inulin-type fructans, which are nondigestible carbohydrates, are capable of decreasing TGs by many mechanisms including 1) decreased hepatic lipogenesis, 2) increased muscle lipoprotein lipase activity and 3) increased short chain fatty acids by bacterial fermentation of carbohydrates which is also capable of inhibiting TG synthesis [16]. In a human study, adding 10 g of high-performance inulin into a moderately high-carbohydrate, low-fat diet in 8 healthy subjects decreased the TG level by inhibiting hepatic lipogenesis [17]. A meta-analysis that included 15 studies concluded that the intake of inulin-type fructans (average amount of inulin was 14.2 g/day [range 4–32]) was associated with a significant decrease in the TG level by 15.05 mg/dl [18]. However, the triglyceride-lowering effect of inulin is inconsistent among trials and is still inconclusive [16, 18, 19]. Thus, the phytosterols and inulin-enriched soymilk may be an alternative to supplement the diet to improve both LDL-c and non-HDL-c to reduce the risk of cardiovascular disease.

Much data confirmed the effects of phytosterols or inulin-enriched products on the lipid profile in many ethnicities but this effect among the Thai population lacks data. This research aims to confirm the effect of phytosterols and inulin on the lipid profile among the Thai population after ingesting phytosterols and inulin-enriched soymilk for 8 weeks.

## Methods

### Study design

This was a double-blinded, randomized-controlled trial that enrolled 240 adult volunteers from the outpatient department and the Endocrinology and Metabolism Unit of Songklanagarind Hospital at Prince of Songkla University from 28 May 2013 to 5 June 2014. The inclusion criteria were 1) subjects who were 18 years old or older and 2) subjects whose LDL-c levels were 130 mg/dl or higher. None of patients were taking statins. The exclusion criteria were 1) subjects who had established cardiovascular disease, 2) subjects who had any type of diabetes mellitus, 3) subjects who had gastrointestinal dysmotility, 4) subjects who had abnormal gastrointestinal digestion or absorption, 5) subjects who had an allergy to soymilk and 6) subjects who had secondary hyperlipidemia such as hypothyroidism, nephrotic syndrome or hepatic disease. Withdrawal or termination criteria were 1) subject’s intention to withdraw, 2) subjects loss to follow-up, 3) elevated transaminase enzymes >3 fold of the upper normal limits, 4) reduced glomerular filtration rate ≥1 stage by the Kidney Disease Outcomes Quality Initiative criteria, 5) subjects unable to tolerate adverse events from soymilk products and 6) compliance of subjects less than 80 % per visit. This study was approved by the Songklanagarind Hospital ethics committee. Written informed consents were obtained before screening and randomization.

The study subjects were randomly assigned by a third-party into the study group which received phytosterols and inulin-enriched soymilk or into the control group which received standard soymilk. The simple random sampling was used to generate a number sequence by the sponsor and the number sequence was given for randomization with an allocation ratio of 1:1. The intervention assignments were concealed to both subjects and clinical staff before randomization. All subjects were given instructions to drink soymilk products twice daily, once in the morning and once in the evening. Subjects who consumed soymilk products less than 80 % were determined as non-compliance.

### Soymilk products

The phytosterols and inulin-enriched soymilk (UHT SOY MILK WITH PHYTOSTEROL; DNA®, Thai FDA food registration number 60-1-05841-2-0089) and standard soymilk in this study were supplied by Dairy Plus Company Limited. A serving unit of both soymilk products contained 180 ml of soymilk, 4.5 g of fat, 5 g of protein and 12 g of carbohydrate. In addition, a serving unit of the phytosterols and inulin-enriched soymilk contained 1 g of phytosterols and 5 g of inulin. The components of the soymilk products are shown in Tables 1 and 2.

**Table 1:** Macronutrient composition of both soymilk products

Component	Percentage (%)	
	Phytosterols and inulin-enriched soymilk	Standard soymilk
- Soy milk	93.3	93.3
- Inulin	3	0
- Sugar	2.5	2.5
- Calcium	0.7	0.7
- Oat flour	0.03	0.03
- Vitamin complex	0.03	0.03
- Green Tea extract	0.015	0.015
- Wheat Grass extract	0.0005	0.0005
- Phytosterols	0.55 (100 %)	0
o Total sterol esters	91 %	
o Free sterols	6 %

**Table 2:** Sterol composition of UHT SOY MILK WITH PHYTOSTEROL; DNA®

Sterol composition^a^ (as free sterols)	By GC, rel. area (%)
Cholesterol	0.0 – 2.0
Brassicasterol	2.0 – 6.0
Campesterol	20.0 – 29.0
Campestanol	0.0 – 6.0
Stigmasterol	12.0 – 23.0
Beta-sitosterol	42.0 – 55.0
Sitostanol	0.0 – 2.5
D5-Avenasterol	0.0 – 4.0
D7-stigmastenol	0.0 – 2.0
D7-Avenasterol	0.0 – 2.0
Other sterols	0.0 – 5.0

^a^ according Food Chemical Codex Monograph

### Data collection at baseline before randomization

Subjects who met the inclusion criteria at the screening visit proceeded to history taking and blood pressure and anthropometry measurements that included body mass index (BMI), waist circumference (WC) and hip circumference (HC) by well-trained personnel. Blood samples for laboratory testing were drawn after fasting for 12 h at screening. All subjects were requested by a nurse to record their eating and exercise behaviors through a 7-day diet and physical activity questionnaire for 1 week before randomization (week 0).

### Follow-up data after randomization

At randomization, all subjects were given guidance to change their eating and exercise behaviors and record them in the 7-day diet and physical activity questionnaire. All subjects had to follow-up at the clinic every 2 weeks for 8 weeks. At each follow-up, all subjects were interviewed for adverse events and underwent a related physical examination. The blood pressure and anthropometry measurements were also taken every visit. Blood samples for biochemistry testing were drawn after fasting for 12 h at weeks 2, 4, 6 and 8. The 75 g oral glucose tolerance tests were conducted at the randomization time (week 0).

### Anthropometry measurements

Well-trained personnel measured the WC and HC. The waist circumference was measured at midline between the lower ribs and the iliac crest during expiration. The hip circumference was measured at the widest part of the hip. The BMI was computed from body weight and height and the waist-hip ratio was computed from the WC and HC measurements.

### Biochemistries

Venous blood samples were drawn between 08:00 and10:00 after a 12-h fast. Serum aliquots were stored at room temperature and transported to the laboratory for testing on the same day. Fasting plasma glucose (FPG), total cholesterol (TC), TG, HDL-c and LDL-c levels were measured by enzymatic *in vitro* assay for direct determination using a MODULAR P800 analyzer. The interassay coefficients of variation of the fasting plasma glucose, total cholesterol, triglyceride, HDL-c and LDL-c were 1.48-1.52 %, 1.57-1.59 %, 1.62-1.71 %, 2.22-2.35 % and 1.96-2.19 %, respectively.

### Adverse events

The subjects were asked every 2 weeks about adverse events including abdominal pain, bloating, nausea, vomiting and increased frequency of defecation or diarrhea when they visited the clinic. Transaminase enzymes and renal function tests were performed every 2 weeks to monitor for any serious adverse events. A serious adverse event was defined by an adverse event which led to withdrawal or termination of the patient from the study.

### Outcomes

Primary outcomes were 1) to compare the LDL-c levels of subjects before and after consumption of the phytosterols and inulin-enriched soymilk for 8 weeks and 2) to compare the LDL-c levels in subjects who consumed the phytosterols and inulin-enriched soymilk to the subjects who consumed standard soymilk. Secondary outcomes were to compare the TC, TG and HDL-c levels in subjects who consumed phytosterols and inulin-enriched soymilk to the subjects who consumed standard soymilk. The potential confounders on the change in the lipid profile included age, sex, BMI and hypertension.

### Sample size calculation

The sample size of the study was calculated using the formula illustrated below.

$$ \begin{array}{l}\mathrm{n}\ \left(\mathrm{pairs}\right)\kern2.5em  = 2{\left[\left({\mathrm{Z}}_{\upalpha} + {\mathrm{Z}}_{\upbeta}\right)\mathrm{S}\mathrm{D}\right]}^2/{\mathrm{D}}^2\\ {}\kern8.5em  = 2{\left[\left(1.96+0.84\right)26.3\right]}^2/95.0625\\ {}\kern8.5em  = 114\ \mathrm{pairs}\end{array} $$

$$ 2\hbox{--} \mathrm{tailed}\ \mathrm{study}\upalpha =0.05,\ {\mathrm{Z}}_{\upalpha}=1.96,\upbeta =0.20,\ {\mathrm{Z}}_{\upbeta}=0.84 $$

To calculate all variables in the formula, we used data referring to the Weidner, C. et al. study [20]. The mean LDL-c level in subjects who consumed phytosterols and inulin-enriched soymilk for 8 weeks decreased from 164 ± 25 mg/dl to 152.9 ± 24 mg/dl while the mean LDL-c level in subjects who consumed standard soymilk decreased from 160 ± 25 mg/dl to 159.5 ± 28.5 mg/dl. The mean difference of the LDL-c between the groups was 9.75 mg/dl.

$$ {\mathrm{D}}^2=\kern1em {(9.75)}^2=\kern1em 95.0625 $$

$$ \mathrm{S}\mathrm{D}=\sqrt{pooled\kern0.5em  variance} $$

$$ =\sqrt{\frac{\left[\left({\mathrm{n}}_1\hbox{-} 1\right)S{D}_1^2+\left({\mathrm{n}}_2\hbox{-} 1\right)S{D}_2^2\right]}{{\mathrm{n}}_1+{\mathrm{n}}_2\hbox{-} 2}} $$

$$ =\sqrt{\frac{19494+13824}{48}} $$

$$ = 26.3\ \mathrm{mg}/\mathrm{dl} $$

The dropout rate was set to be at least 5 %, thus the total number of subjects in this study was calculated to be approximately 240 subjects (120 subjects in each arm)

### Statistical analysis

Descriptive data were showed in mean, median, standard deviation (SD) and range (min,max). The likelihood ratio test or Fisher’s exact test was used to identify differences between the baseline characteristics which were categorical data and the Mann–Whitney *U* test was used in case of continuous data. The statistical analysis to determine the difference of LDL-c and other lipids between the study groups used the Mann–Whitney *U* test. The statistical analysis to determine the difference of LDL-c between pre- and post-intervention in each study group used the Wilcoxon signed-rank test. Multiple linear regression was used to adjust potential confounders including age, sex, BMI and hypertension, using the baseline LDL-c value as a covariate. A change-in-estimation criterion with a cut-off of 10 % was used to identify the confounders [21]. Pearson correlation was used to determine the correlation between baseline LDL-c and percent LDL-c change. Pearson Chi-square was used to determine the relative risk of adverse events between groups. A *p*-value <0.05 indicated statistical significance. R-3.1.2 for Windows was used for the data analyses.

## Results

**Fig. 2:**
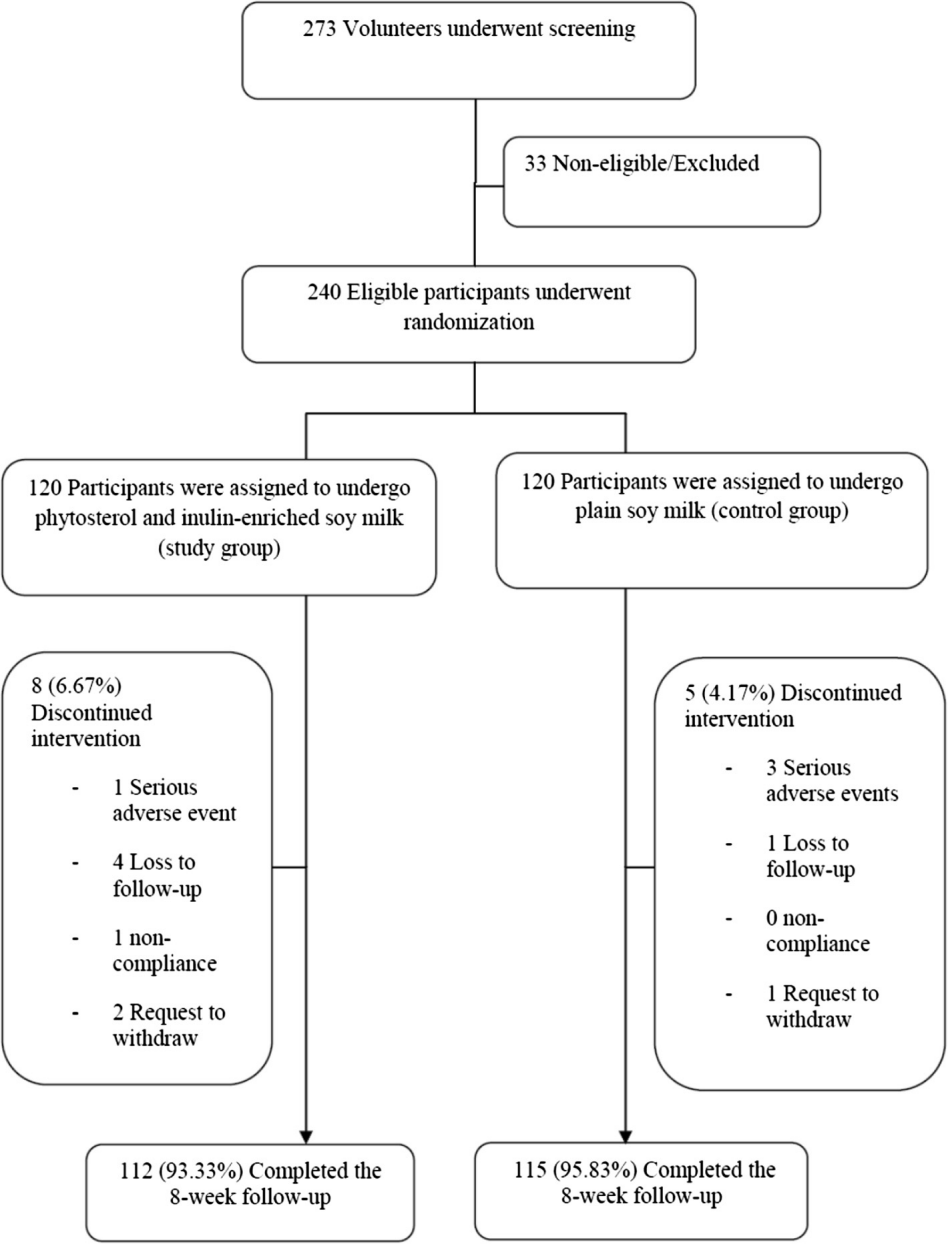
CONSORT flow diagram

Two hundred and seventy-three volunteers were screened. The number of subjects who met the inclusion criteria and enrolled into the study was 240. The enrolled subjects were 1:1 randomized into the study group or the control group which put 120 subjects in each group. Thirteen subjects (5.42 %) withdrew from the study; 4 subjects had serious adverse events, 5 subjects were loss to follow-up, 1 subject was noncompliant and 3 subjects requested withdrawal from the study because of pregnancy, anterior cruciate ligament surgery and the inconvenience of drinking a soymilk product. Two hundred and twenty-seven subjects (94.58 %) were included into the primary outcome analysis (Fig. 2).

Demographic and baseline characteristics of the subjects are shown in Table 3. Subjects were predominantly female (85 %); 57.5 % of the subjects were government employees and 19.16 % of the subjects were university employees. Cardiovascular risk factors of the subjects in the study were 91.67 % of the subjects never smoked, 3.75 % of the subjects reported they had hypertension, 9.09 % of the subjects reported dyslipidemia and 2.5 % of the subjects reported a family history of cardiovascular disease. The median age of the subjects was 47.5 (18,71) years old, mean BMI was 24.20 ± 3.48 kg/m^2^, median WC was 82.5 (60,122) cm, median systolic blood pressure was 117 (87,176) mmHg and median diastolic blood pressure was 72 (36,117) mmHg, median FPG was 90 (71,114) mg/dl and the median LDL-c level was 165 (130,254) mg/dl. There were no differences in the demographic and baseline characteristic data between the study group and the control group except the report of dyslipidemia in the study group was higher than the control group (12.5 % vs 4.17 %, *p* = 0.033).

**Table 3:** Comparison of baseline characteristics, biochemical tests and lipid profiles between groups (*n* = 240)

Baseline characteristics		Number (%) or mean ± SD or median (min,max)		*p*-value
		Study group (*n* = 120)	Control group (*n* = 120)	
Sex^b^				0.857
	Female	101 (84.2)	103 (85.8)	
	Male	19 (15.8)	17 (14.2)	
age (year), median (min,max)^d^		48 (24,71)	46 (18,68)	0.75
Career^a^				0.556
	Government employees	73 (60.8)	65 (54.2)	
	State enterprise employees	1 (0.8)	2 (1.7)	
	Company employees	5 (4.2)	2 (1.7)	
	Self-employed	4 (3.3)	2 (1.7)	
	Servants	12 (10.0)	18 (15.0)	
	Pensioners	4 (3.3)	5 (4.2)	
	Students	0	1 (0.8)	
	University employees	21 (17.5)	25 (20.8)	
Smoking^a^				0.635
	Never smoked	109 (92.4)	111 (92.5)	
	Ex-smoker	4 (3.4)	6 (5.0)	
	Currently smoked	5 (4.2)	3 (2.5)	
Alcohol drinking^b^		42 (36.5)	33 (29.7)	0.323
Alcohol drinking in past 12 months^a^				0.310
	Every week	2 (6.5)	4 (16.0)	
	1-3 times/month	5 (16.1)	5 (20.0)	
	Less than once a month	24 (77.4)	16 (64.0)	
Familial history of CVD^b^		3 (2.5)	3 (2.5)	1.00
Hypertension^b^		2 (1.7)	7 (5.8)	0.171
Dyslipidemia^b^		15 (12.5)	5 (4.2)	0.033*
Body mass index (BMI) (kg./m^2^)^c^		23.81 ± 3.26	24.58 ± 3.66	0.085
Waist circumference (WC) (cm)		81 (65,122)	84.25 (60,112)	1.430
Hip Circumference (cm)^c^		96.70 ± 6.87	97.51 ± 7.10	0.373
Waist hip ratio (WHR)^d^		0.85 (0.70,0.96)	0.87 (0.73,1.03)	0.322
Systolic blood pressure (mmHg)^d^		115.50 (92,175)	118 (87,176)	0.409
Diastolic blood pressure (mmHg)^d^		72 (36,110)	72 (52,117)	0.955
Fasting plasma glucose (mg/dl)^d^		90 (75,112)	90 (71,114)	0.146
Total cholesterol (mg/dl)^c^		244.07 (28.92)	239.26 (27.19)	0.238
Triglyceride (mg/dl)^d^		99.5 (45,295)	97.5 (39,399)	0.904
HDL Cholesterol (mg/dl)^c^		63.51 (14.96)	60.00 (13.57)	0.207
LDL Cholesterol (mg/dl)^d^		165 (132,254)	165 (130,243)	0.385
Creatinine (mg/dl)^d^		0.69 (0.42,1.22)	0.70 (0.51,1.23)	0.258
AST (U/L)^d^		19 (7,85)	20 (13,66)	0.271
ALT (U/L)^d^		16 (8,75)	18 (9,81)	0.106

*p < .05, a = likelihood ratio test, b = Fisher’s exact test, c = independent *t*-test, d = Mann–Whitney *U* test

**Fig. 3:**
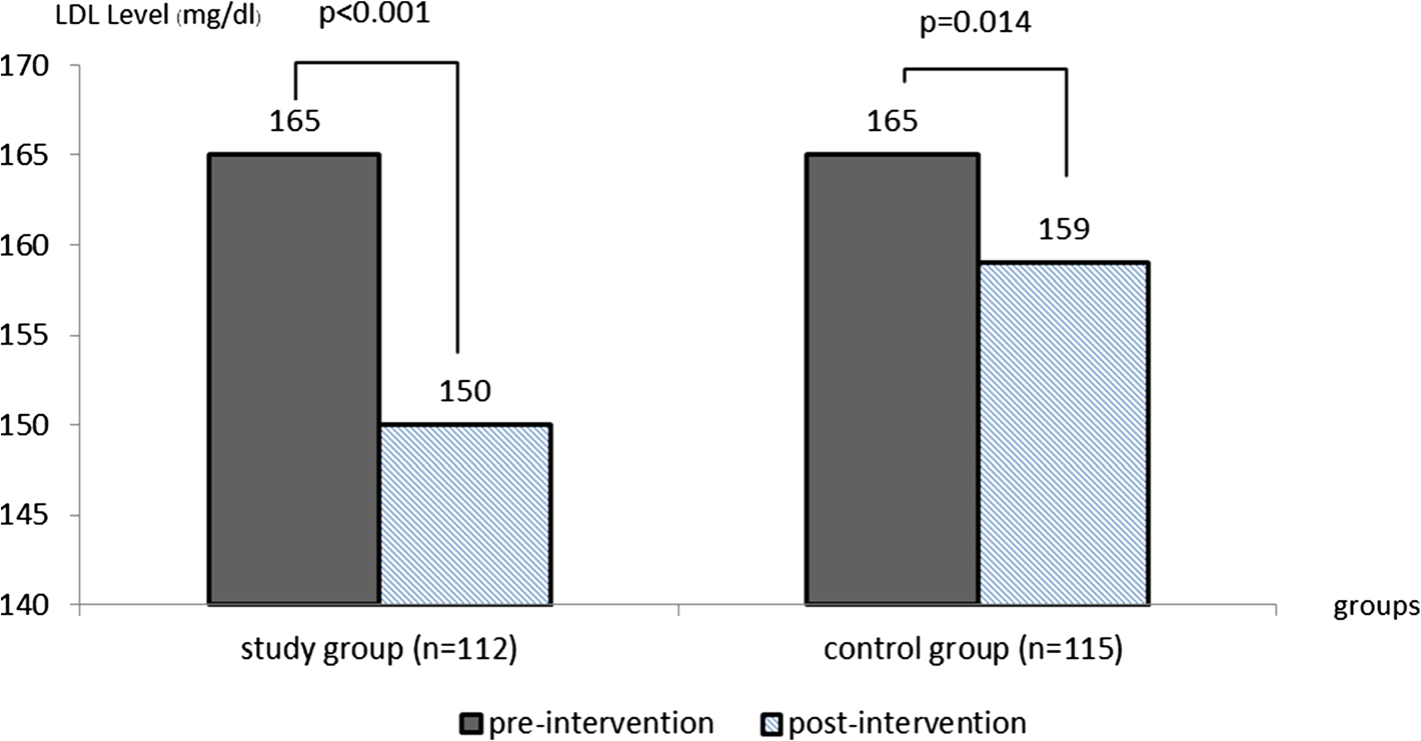
Pre- and post-intervention LDL-c levels between the study and control groups at weeks 0 and 8 by Wilcoxon signed-rank test (*n* = 227)

**Fig. 4:**
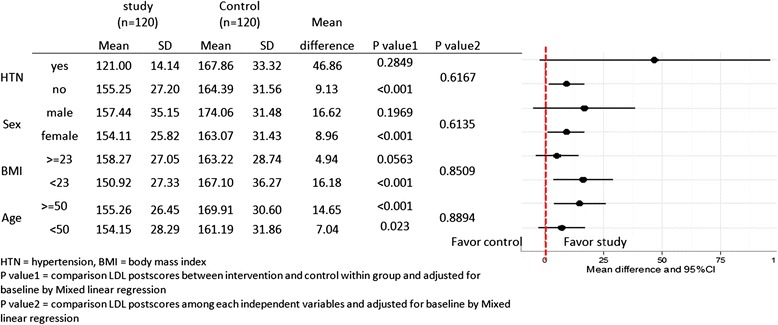
Subgroup-analysis determined the mean difference of LDL-c reduction between study and control groups based on patients’ hypertension status, sex, BMI and age (*n* = 240)

At the end of the study, the median LDL-c levels decreased significantly from 165 (132,254) mg/dl to 150 (105,263) mg/dl in the study group (*p* < 0.001) and from 165 (130,243) mg/dl to 159 (89,277) mg/dl in the control group (*p* = 0.014) (Fig. 3). The biweekly lipid profiles in both groups are shown for comparison in Table 4. The difference in the LDL-c levels between the groups started to show at the second week and it persisted until the end of the study (p < 0.05). The percent LDL-c reduction in the study group was significantly greater than the control group (−10.03 % (−37.07, 36.00 %) vs −1.31 % (−53.40, 89.73 %), *p* < 0.001) (Table 5) and the LDL-c reduction efficacy of both soymilk products was also independent to potential confounders including age, sex, BMI and hypertension after they were adjusted (Additional file 1). Subgroup analysis showed trends of LDL-c reduction were not different between subgroups defined by 1) with or without hypertension, 2) male or female, 3) normal weight or over weight (for the Asian population) and 4) younger or older than 50 years (Fig. 4).

**Table 4:** Comparison of total cholesterol, triglyceride, HDL-c and LDL-c between the study and control groups at weeks 0, 2, 4, 6 and 8 (*n* = 240)

		Median (min,max)		*p*-value
		Study group (*n* = 120)	Control group (*n* = 120)	
Total cholesterol	Week 0	239.5 (191,368)	235 (178,317)	0.238
	Week 2	223 (162,336)	235 (167,305)	0.065
	Week 4	228 (164,319)	229 (166,316)	0.203
	Week 6	225 (118,308)	234 (151,309)	0.011*
	Week 8	223.50 (173,335)	232 (147,319)	0.144
Triglyceride	Week 0	99.5 (45,295)	97.5 (39,399)	0.904
	Week 2	94.0 (41,309)	99.0 (30,364)	0.782
	Week 4	92 (34,330)	100 (30,423)	0.438
	Week 6	94.5 (41,336)	97 (41,481)	0.512
	Week 8	95.5 (38,277)	107 (41,304)	0.494
HDL cholesterol	Week 0	61 (34,109)	59 (21,97)	0.207
	Week 2	60 (31,99)	58 (32,93)	0.344
	Week 4	62 (36,102)	58 (28,92)	0.131
	Week 6	63 (33,96)	60 (29,96)	0.082
	Week 8	60.5 (35,103)	57 (27,97)	0.174
LDL cholesterol	Week 0	165 (132,254)	165 (130,243)	0.385
	Week 2	153 (95,256)	165 (91,235)	0.032*
	Week 4	150.50 (91,234)	160 (97,244)	0.024*
	Week 6	151 (96,223)	163 (93,234)	0.003**
	Week 8	150 (105,263)	159 (89,277)	0.009**

Mann–Whitney *U* test, *p < .05 ** *p* < .01

**Table 5:** Comparison of percentage changes in the total cholesterol, triglyceride, HDL-c and LDL-c levels between the study and control groups at week 8 (*n* = 240)

	Median (min,max)		*p*-value*
	Study group (*n* = 120)	Control group (*n* = 120)	
Total cholesterol	−6.60 (−32.35,35.00)	−1.76 (−41.43,34.35)	<0.001
Triglyceride	0.96 (−41.14,128.93)	0.50 (−69.42,176.40)	0.96
HDL Cholesterol	1.53 (−53.85,24.59)	0 (−25.42,57.14)	0.70
LDL Cholesterol	−10.03 (−37.07,36.00)	−1.31 (−53.40,89.73)	<0.001

*Mann–Whitney *U* test

**Fig. 5:**
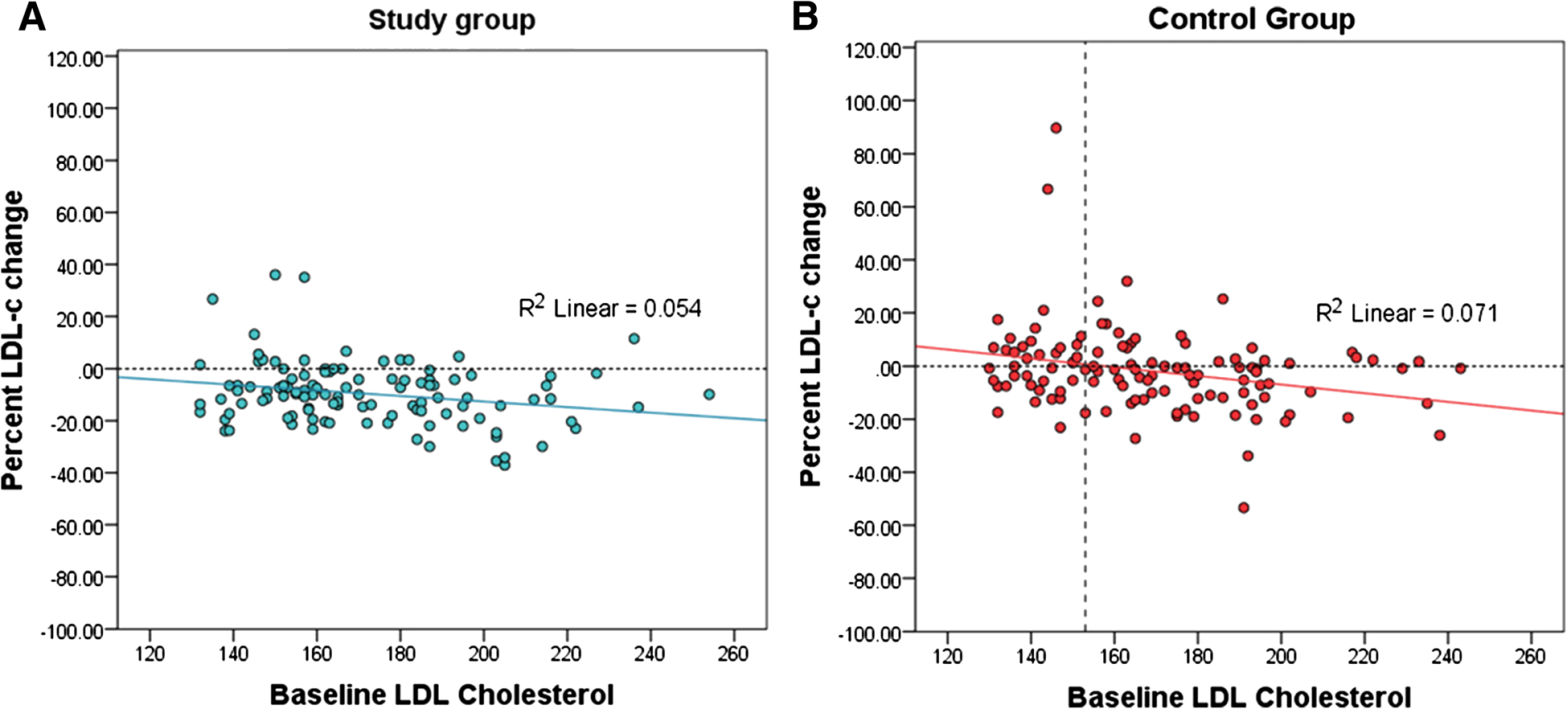
Correlation between baseline LDL-c and percent LDL-c change; (**a**) study group (*n* = 115), (**b**) control group (*n* = 112)

Pearson correlation showed a small but significant correlation between the baseline LDL-c and percent LDL-c change in the study group (*r* = −0.23, *p*-value =0.007). Linear regression analysis was used to determine the LDL-c reduction effect of phytosterols and inulin enriched soymilk and found that for every 1 mg/dl increment of baseline LDL-c, the LDL-c reduction increased by 0.11 % (coefficient = −0.11, *p*-value = 0.014). In the control group, although there was also a small but significant correlation found between the baseline LDL-c and percent LDL-c change (*r* = −0.27, p-value =0.002), it should be noted that the LDL-reduction effect of standard soymilk was seen only if the baseline LDL-c was over 153 mg/dl while the standard soymilk would increase LDL-c level among the subjects with lower baseline LDL-c. This pattern is explained by the ‘regression to the mean’ phenomenon (Fig. 5).

The phytosterols and inulin-enriched soymilk also significantly reduced the TC level compared to standard soymilk. The percentage of TC reduction in the study group was 6.60 % while it was only 1.76 % in the control group (*p* < 0.001). The TG level did not significantly change in either group which increased by 0.96 % in the study group and 0.50 % in the control group (*p* = 0.96). The HDL-c level also did not significantly change in either group which increased by 1.53 % in the study group and unchanged in the control group (*p* = 0.70) (Table 5).

In both study and control groups, the FPG levels were similar to the baseline levels during the entire study period and they were not different between the groups (Additional file 2). Thirty-one percent of subjects in the study group had adverse events while 23.3 % of subjects in the control group had adverse events (relative risk = 1.33 [0.87-2.03, 95 % CI]). There were four serious adverse events that occurred in the study included one severe diarrhea in the study group and another in the control group, one severe bloating in the control group and one hepatitis (defined by transaminase enzymes that were 3 fold higher than the upper normal limits) in the control group. The most common adverse events was bloating (overall 71.56 %) which seemed to be more common in the study group (76.92 %) than the control group (64.72 %) and it seemed to more persistent in the study group (additional file 3). The adverse events in each group are shown in Table 6.

**Table 6:** Number of subjects having adverse events from total visits and the adverse events categorized by severity in the study and control groups

	Number (%)				
	Study group (*n* = 116)^a^	Control group (*n* = 120)	Total (*n* = 236)	RR [95 % CI]	*p*-value
Subjects had adverse events, person	36 (31.0)	28 (23.3)	64 (27.12)	1.33 [0.87-2.03]	0.177^b^
Actual adverse events, event	65 (100)	51 (100)	116 (100)		
Serious adverse events					
Bloating	0 (0)	1 (1.96)	1 (0.86)		
Hepatitis	0 (0)	1 (1.96)	1 (0.86)		
Severe diarrhea	1 (1.54)	1 (1.96)	2 (1.72)		
Non-serious adverse events					
Bloating	50 (76.92)	33 (64.72)	83 (71.56)		
Abdominal cramp	0 (0)	1 (1.96)	1 (0.86)		
Nausea	0 (0)	4 (7.84)	4 (3.45)		
Vomiting	1 (1.54)	1 (1.96)	2 (1.72)		
Increase defecation	2 (3.08)	0 (0)	2 (1.72)		
Diarrhea	4 (6.15)	4 (7.84)	8 (6.90)		
Constipation	2 (3.08)	2 (3.92)	4 (3.45)		
Other	5 (7.69)	3 (5.88)	8 (6.90)		

^a^3 subjects loss to follow up before the week 2, 1 subject requested to withdraw due to pregnancy

^b^Pearson Chi-square

## Discussion

The present study confirmed LDL-c reduction with 1 g phytosterols and 5 g inulin-enriched soymilk twice daily among statin-naïve, mild-to-moderate hypercholesterolemia subjects. The absolute LDL-c reduction efficacy of 2 g/day of phytosterols and 10 g/day of inulin was 8.72 % which was comparable to previous studies. Kriengsinyos W. et al. reported an LDL-c reduction efficacy of 2 g stanol-containing soymilk once a day and postprandially for 6 weeks that was an absolute 8.9 % [22]. The LDL-c reduction efficacy was comparable to either once daily or twice daily administration as long as the total dose of phytosterols was 2 g per day. Two grams daily of phytosterols reduced LDL-c level by 8-14 %, which depended on the initial baseline LDL-c of the subjects. At a higher baseline LDL-c, the benefits of phytosterols were apparently observed [23, 24]. In the present study, LDL-c reduction efficacy of phytosterols and inulin enriched soymilk had a small but significant correlation to baseline LDL-c. Every 1 mg/dl increment of baseline LDL-c affected the LDL-c reduction by an increase of 0.11 %, while a similar correlation also existed in the standard soymilk group but it seemed to be a ‘regression to the mean’ phenomenon. The LDL-c level was significantly lower in the study group from the second week and it was constantly lower in the study group throughout the study period. The action of phytosterols to inhibit intestinal cholesterol absorption seemed to occur instantly after consuming the phytosterols-enriched soymilk. The LDL-c level was also significantly reduced in subjects who consumed the standard soymilk but its effect was trivial. This effect probably occurred because 1) the subjects possibly changed their eating and exercise behaviors after enrollment in the study and 2) soy protein in the standard soymilk had an LDL-c reduction effect [25, 26].

The effect of phytosetrols may be influenced by the types of food carriers. A previous meta-analysis reported that high-fat content food carriers might have a greater effect than low-fat content food carriers [23]. One previous study demonstrated the LDL-c lowering effects of phytosterols incorporated into various food carriers, the absolute LDL-c reductions for yoghurt, hard cheese and fresh cheese were 7.7 %, 10.9 % and 8.6 %, respectively [27]. Many reports demonstrated the LDL-c lowering effects of phytosterols incorporated into low-fat content food carriers such as low-fat yoghurt [28, 29], milk [29], yoghurt drinks [30] and orange juice [31], which varied from 6.4 % to 15.9 %. Hence, the low-fat content food carriers were good matrixes for incorporation of phytosterols. Few studies, including the present one, reported the LDL-c lowering effects of 7-13 % of phytosterols-enriched soymilk on LDL-c which was similar to other low-fat content food carriers [20, 22, 32]. Thus, soymilk also proved to be a good matrix for incorporation of phytosterols.

LDL-c is a well-known important risk factor for cardiovascular disease while every 1 mg of LDL-c reduction relatively decreases the risk of having a coronary event by 1 % [33]. The phytosterols-enriched soymilk, which proved its LDL-c reduction effect, may have other effects to reduce the risk of cardiovascular disease; however, further investigations are required to determine the cardiovascular outcome.

High TG and low HDL-c levels are other risk factors for cardiovascular disease particularly in people with metabolic syndrome or T2DM. Due to the concern that the additional carbohydrate and free fatty acid from both soymilk products might increase the TG levels and lower the HDL-c levels, the effects of both soymilk products on TG and HDL-c levels were determined. In the present study, the phytosterols and inulin-enriched soymilk and standard soymilk had a neutral effect on both TG and HDL-c levels which was in agreement with previous studies [11, 34]. The ESC/EAS guideline also concluded that 2 g daily of phytosterols had little or no effect on TG and HDL-c levels [3]. However, it should be noted that subjects recruited into this study were metabolically lean with low TG and high HDL-c levels and subjects with T2DM were excluded from the study. Thus, we cannot imply that either soymilk product would not increase TG levels in subjects with metabolic syndrome or T2DM.

On the contrary, we expected to see a better TG level in the study group from the inulin effect but the present study could not demonstrate a positive effect from 10 g/day of inulin to decrease TG levels. Letexier et al. demonstrated TG reduction efficacy of 10 g/day of inulin when it was consumed with a low-fat, high-carbohydrate (LF/HC) diet [17]. Consumption of a LF/HC diet increased TG levels in both fasting and postprandial states by elevations of VLDL and chylomicrons in both states [35]. The mechanism of an LF/HC diet to increase TG levels was decreased VLDL clearance from the blood by lipoprotein lipase rather than increased VLDL secretion or *de novo* lipogenesis. The decreased VLDL clearance was possibly due to competition between VLDL and chylomicron remnants on lipoprotein lipase [36]. Thus consuming inulin together with an LF/HC diet might improve TG level by inhibiting carbohydrate absorption, resulting in reduction of chylomicron remnants released by the intestine. In our study, there were no changes in the dietary pattern of a LF/HC diet and there was no monitoring of the dietary pattern, thus the TG reduction effect of the inulin in the present study was unseen. We did not measure the apolipoprotein (Apo) B100 which reflects VLDL production and Apo B48 which reflects chylomicron assembly by the intestine, thus we did not know whether the source of TG in our subjects came from *de novo* lipogenesis or a decrease in VLDL clearance by competition from chylomicron remnants. The baseline TG level might be another important factor for inulin efficacy. Jackson KG et al. demonstrated the effect of 10 g inulin sachets add-on in water, tea, coffee, orange juice, soup, breakfast cereal or yoghurt on TG reduction and concluded that the percent TG change was correlated with the initial TG concentration, particularly in subjects in whom fasting TG levels were greater than 132.8 mg/dl [37]. Since the baseline median TG level in the present study was lower than 100 mg/dl, inulin efficacy was not seen in this study group.

The phytosterols and inulin-enriched soymilk and the standard soymilk did not alter glycemic levels in either the study group or the control group (Supplementary 1). However, the present study did not include diabetic patients into the study, thus we could not determine the effect of soymilk products on glycemic level in diabetic patients.

Occurrences of adverse events in both groups were similar and usually well-tolerated. Bloating was the most common adverse effect in both of the study and control groups, which accounted for 71.56 % of overall adverse events and seemed to be more common in the phytosterols and inulin-enriched soymilk group. Neither of the soymilk products contained lactose so the possible cause of bloating was soy protein intolerance. Since inulin is a non-digestible carbohydrate and its fermentation possibly causes the intestinal gas which may explain why the bloating was more common in the study group [38]. There were 4 serious adverse events, one intolerable diarrhea in the study group and 3 events in the control group that included an intolerable bloating, an intolerable diarrhea and hepatitis. Therefore, the phytosterols and inulin-enriched soymilk seemed to be as equally safe as the standard soymilk.

The strengths of the present study were 1) the subjects included in the study were randomized well as indicated by the comparable baseline characteristics in both groups, 2) the soymilk products were blinded to both subjects and investigators, 3) overall 94.58 % of the data were complete and 4) all subjects enrolled into the study were statin-naïve, so the present study proved the pure effect of phytosterols. The present study also encouraged the role of phytosterols-enriched soymilk to improve the LDL-c level among people who live in an urban area.

Limitations of the present study were 1) all subjects enrolled into the study were statin-naïve, thus we could not determine additional or synergistic effect of phytosterols on those who had prior statin-use, 2) the aim of the study was to determine the short term effect on lipid profile, so the benefit on cardiovascular outcome was undetermined by the present study and 3) the homogeneity of subjects who were non-DM and metabolically lean limited the application of the study to T2DM or metabolic syndrome subjects.

## Conclusions

Consumption of soymilk containing 2 g of phytosterols and 10 g of inulin reduced LDL-c and TC better than standard soymilk. The LDL-c lowering efficacy was clearly seen from the second week. The phytosterols and inulin-enriched soymilk had no effect on TG, HDL-c and glycemic levels among the non-diabetic subjects and it was as safe as the standard soymilk. The phytosterols and inulin-enriched soymilk is an alternative way to reduce LDL-c levels to reduce the risk of cardiovascular disease.

## Additional files

Additional file 1:
**The difference of percent changes of the total cholesterol, triglyceride, HDL-c and LDL-c levels between groups before and after potential confounders were adjusted (n = 240).** (DOCX 14 kb) Additional file 2:
**Comparison of fasting plasma glucose level between the study and control groups at weeks 0, 2, 4, 6 and 8 by Mann–Whitney** ***U*** **test.** (DOCX 50 kb) Additional file 3:
**The relative risk of persistent bloating in the study group compared to the control group.** (DOCX 17 kb)

## Abbreviations

LDL-c: Low-density lipoprotein cholesterol
NPC1L1: Niemann-Pick C1-like 1
TG: Triglyceride
HDL-c: High-density lipoprotein cholesterol
VLDL: Very low-density lipoprotein
BMI: Body mass index
WC: Waist circumference
HC: Hip circumference
FPG: Fasting plasma glucose
TC: Total cholesterol
CVD: Cardiovascular diseases
LF/HC: Low-fat, high-carbohydrate
T2DM: Type 2 diabetes mellitus
