# Supplementary material for: Effect of phytosterols and inulin-enriched soymilk on LDL-cholesterol in Thai subjects: a double-blinded randomized controlled trial
Source: Lipids Health Dis. 2015 Nov 9;14:146. doi: 10.1186/s12944-015-0149-4 (PMC4640379; doi:10.1186/s12944-015-0149-4)
Supplement: Additional file 1: — The difference of percent changes of the total cholesterol, triglyceride, HDL-c and LDL-c levels between groups before and after potential confounders were adjusted (n = 240). (DOCX 14 kb) [file 12944_2015_149_MOESM1_ESM.docx]

**Additional file1.** The difference of percent changes of the total cholesterol, triglyceride, HDL-c and LDL-c levels between groups before and after potential confounders were adjusted (n=240)

|  | Mean (SD) | | Difference between groups  [95%CI] | Adjusted coefficient† | p-value |
| --- | --- | --- | --- | --- | --- |
|  | Study group (n=120) | Control group (n=120) |  |  |  |
| TC | -14.7 (24.8) | -4.0 (24.2) | -10.7 (-17.1, -4.3) | -10.7, (-17.3, -4.1) | 0.001 |
| TG | 3.63 (34.8) | 0.83 (49.5) | 2.8 (-13.9, 8.4) | 3.7 (-7.79, 15.28) | 0.524 |
| HDL-c | -0.58 (8.4) | -0.14 (7.5) | -0.45 (-1.63, 2.53) | -0.64, (-2.77, 1.49) | 0.553 |
| LDL-c | -17.2 (21.5) | -4.2 (26.9) | -12.9 (-19.3, -6.6) | -12.7 (-19.3, -6.2) | <0.001 |
| †multiple linear regression adjusted for age, sex, BMI and HTN  § independent t-test,  *p < .05, ** p < .01, ***p<0.001. | | | | | |

The comparison change scores of TG, TC, HDL and LDL were tested by independent test. The mean (SD) of each lipid component was used instead of the median to meet the assumption before multiple linear regression model was performed to adjust for possible confounders including age, sex, BMI and HTN. Change-in-estimation criterion with a cut-off of 10% was used to identify the confounders. When we compared crude coefficient of percent LDL-c reduction (12.9%) to adjusted coefficient (12.7%), the difference was only 2% which meant that the potential confounders had no effect on percent LDL-c reduction between the study and control groups.
