# Supplementary material for: Effect of phytosterols and inulin-enriched soymilk on LDL-cholesterol in Thai subjects: a double-blinded randomized controlled trial
Source: Lipids Health Dis. 2015 Nov 9;14:146. doi: 10.1186/s12944-015-0149-4 (PMC4640379; doi:10.1186/s12944-015-0149-4)
Supplement: Additional file 2: — Comparison of fasting plasma glucose level between the study and control groups at weeks 0, 2, 4, 6 and 8 by Mann–Whitney U test. (DOCX 50 kb) [file 12944_2015_149_MOESM2_ESM.docx]

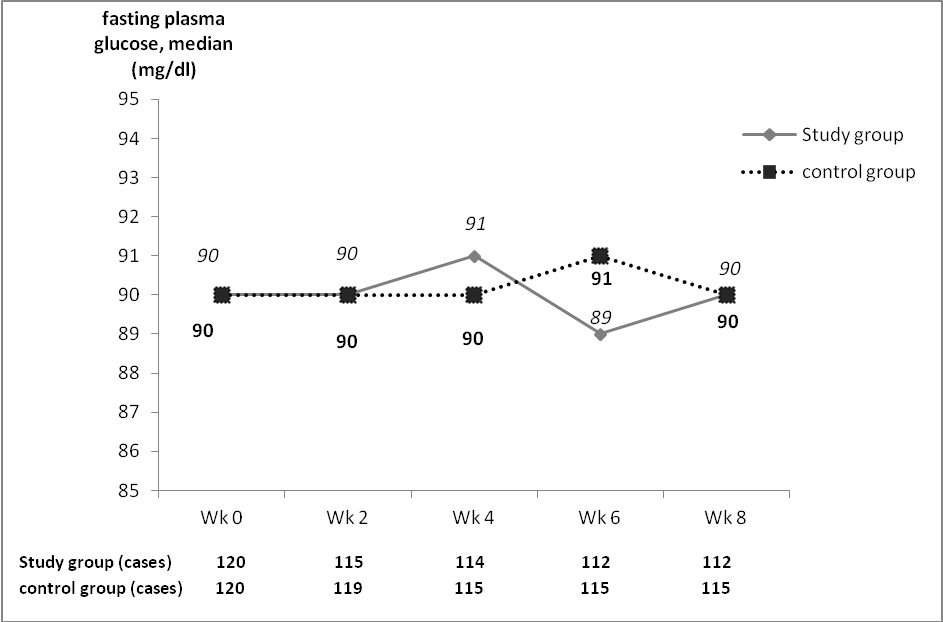


**Additional file 2**. Comparison of fasting plasma glucose level between the study and control groups at weeks 0, 2, 4, 6 and 8 by Mann-Whitney U test
