# Supplementary material for: Effect of phytosterols and inulin-enriched soymilk on LDL-cholesterol in Thai subjects: a double-blinded randomized controlled trial
Source: Lipids Health Dis. 2015 Nov 9;14:146. doi: 10.1186/s12944-015-0149-4 (PMC4640379; doi:10.1186/s12944-015-0149-4)
Supplement: Additional file 3: — The relative risk of persistent bloating in the study group compared to the control group. (DOCX 17 kb) [file 12944_2015_149_MOESM3_ESM.docx]

|  | | Number (%) | | |  |
| --- | --- | --- | --- | --- | --- |
|  | | Study group^§^ (n=116) | Control group (n=120) | Total | RR [95% CI] |
| Persistency of bloating, person (%) | none | 88 (47.1) | 99 (52.9) | 187 (100) | 1.0 (ref.) |
|  | 1-2vistits | 20 (54.1) | 17 (45.9) | 35 (100) | 1.26 [0.70-2.28] |
|  | >3visits | 8 (66.7) | 4 (33.3) | 12 (100) | 2.15 [0.67-6.90] |

**Additional file 3.**  The relative risk of persistent bloating in the study group compared to the control group.
